# Quantifying the effects of hydrogen on carbon assimilation in a seafloor microbial community associated with ultramafic rocks

**DOI:** 10.1038/s41396-021-01066-x

**Published:** 2021-07-26

**Authors:** Ömer K. Coskun, Aurèle Vuillemin, Florence Schubotz, Frieder Klein, Susanna E. Sichel, Wolfgang Eisenreich, William D. Orsi

**Affiliations:** 1 Department of Earth and Environmental Sciences, Ludwig-Maximilians-Universität, Munich, Germany; 2 MARUM Center for Marine Environmental Sciences, University of Bremen, Bremen, Germany; 3 Woods Hole Oceanographic Institution, Woods Hole, MA USA; 4 Departamento de Geologia e Geofísica/LAGEMAR–Universidade Federal Fluminense-Brazil, Niterói, RJ Brazil; 5 Department of Chemistry, Bavarian NMR Center–Structural Membrane Biochemistry, Technische Universität München, Garching, Germany; 6 GeoBio-CenterLMU, Ludwig-Maximilians-Universität München, Munich, Germany; 7 Present Address: GFZ German Research Centre for Geosciences, Helmholtz Centre Potsdam, Potsdam, Germany

**Keywords:** Microbial ecology, Water microbiology, Microbial biooceanography, Microbiome, Biogeochemistry

## Abstract

Thermodynamic models predict that H_2_ is energetically favorable for seafloor microbial life, but how H_2_ affects anabolic processes in seafloor-associated communities is poorly understood. Here, we used quantitative ^13^C DNA stable isotope probing (qSIP) to quantify the effect of H_2_ on carbon assimilation by microbial taxa synthesizing ^13^C-labeled DNA that are associated with partially serpentinized peridotite rocks from the equatorial Mid-Atlantic Ridge. The rock-hosted seafloor community was an order of magnitude more diverse compared to the seawater community directly above the rocks. With added H_2_, peridotite-associated taxa increased assimilation of ^13^C-bicarbonate and ^13^C-acetate into 16S rRNA genes of operational taxonomic units by 146% (±29%) and 55% (±34%), respectively, which correlated with enrichment of H_2_-oxidizing NiFe-hydrogenases encoded in peridotite-associated metagenomes. The effect of H_2_ on anabolism was phylogenetically organized, with taxa affiliated with *Atribacteria*, *Nitrospira*, and Thaumarchaeota exhibiting the most significant increases in ^13^C-substrate assimilation in the presence of H_2_. In SIP incubations with added H_2_, an order of magnitude higher number of peridotite rock-associated taxa assimilated ^13^C-bicarbonate, ^13^C-acetate, and ^13^C-formate compared to taxa that were not associated with peridotites. Collectively, these findings indicate that the unique geochemical nature of the peridotite-hosted ecosystem has selected for H_2_-metabolizing, rock-associated taxa that can increase anabolism under high H_2_ concentrations. Because ultramafic rocks are widespread in slow-, and ultraslow-spreading oceanic lithosphere, continental margins, and subduction zones where H_2_ is formed in copious amounts, the link between H_2_ and carbon assimilation demonstrated here may be widespread within these geological settings.

## Introduction

The oxidation of molecular hydrogen (H_2_) is an important source of bioavailable energy in anoxic environments, and H_2_ represents a key metabolic intermediate in anaerobic syntrophy [1, 2]. Recently, aerobic H_2_ oxidation was discovered to be widespread amongst microbial “dark matter” [3–5], with many aerobic microbial groups being capable of scavenging trace concentrations of atmospheric H_2_ as an energy source [6]. However, the effects of H_2_ on carbon utilization rate in marine microbial communities under low-oxygen conditions are poorly understood. The oxidation of H_2_ with O_2_ is predicted to be a thermodynamically favorable energy source for peridotite-associated microbial communities over a wide range of temperatures in ultramafic-hosted systems [7] and thus has the potential to provide important catabolic energy for seafloor-associated communities that live in the vicinity of a geological H_2_ source [7, 8]. Indeed, a linkage between H_2_ and microbial activity has been demonstrated in several high-temperature hydrothermal settings [8–12].

To better understand the effects of H_2_ on carbon anabolism in seafloor microbial communities associated with ultramafic rocks, we used ^13^C quantitative DNA stable isotope probing (qSIP) [13, 14] with ^13^C-labeled bicarbonate, acetate, and formate in H_2_ incubation experiments. The ^13^C DNA-qSIP approach identifies microbial taxa that are synthesizing new ^13^C-labeled DNA from the added ^13^C substrates, which occurs during genome replication [15]. We applied this method to microbial communities associated with partially serpentinized peridotite mylonite from Saint Peter and Saint Paul Archipelago (Arquipélago de São Pedro e São Paulo, Brasil ‘SPSPA’) at the equatorial Mid-Atlantic Ridge.

The SPSPA is mainly composed of strongly deformed, partially serpentinized Mg- and Fe-rich (i.e. ultramafic) rocks [16]. Serpentinization of ultramafic rocks involves the oxidation of ferrous iron in primary minerals to ferric iron in secondary minerals by water which generates abundant H_2_ that can be used to conserve energy by H_2_-oxidizing microbes [17]. During a recent expedition to SPSPA (AL170602) onboard the MV ALUCIA in 2017, geochemical evidence for H_2_ formation was found to be recorded in serpentinized rocks and H_2_ is likely generated today at SPSPA through low-temperature aqueous alteration of peridotite, mechanoradical H_2_ formation, or radiolysis [17], albeit at slow rates. Thus, we incubated partially serpentinized peridotite from SPSPA with ^13^C-bicarbonate, ^13^C-acetate, and ^13^C-formate in incubations with and without H_2_, and then applied qSIP [13, 14] to quantify the effects of H_2_ on ^13^C-substrate assimilation by specific operational taxonomic units (OTUs) that were associated with the ultramafic rocks.

## Material and methods

### Sampling

**Fig. 1:**
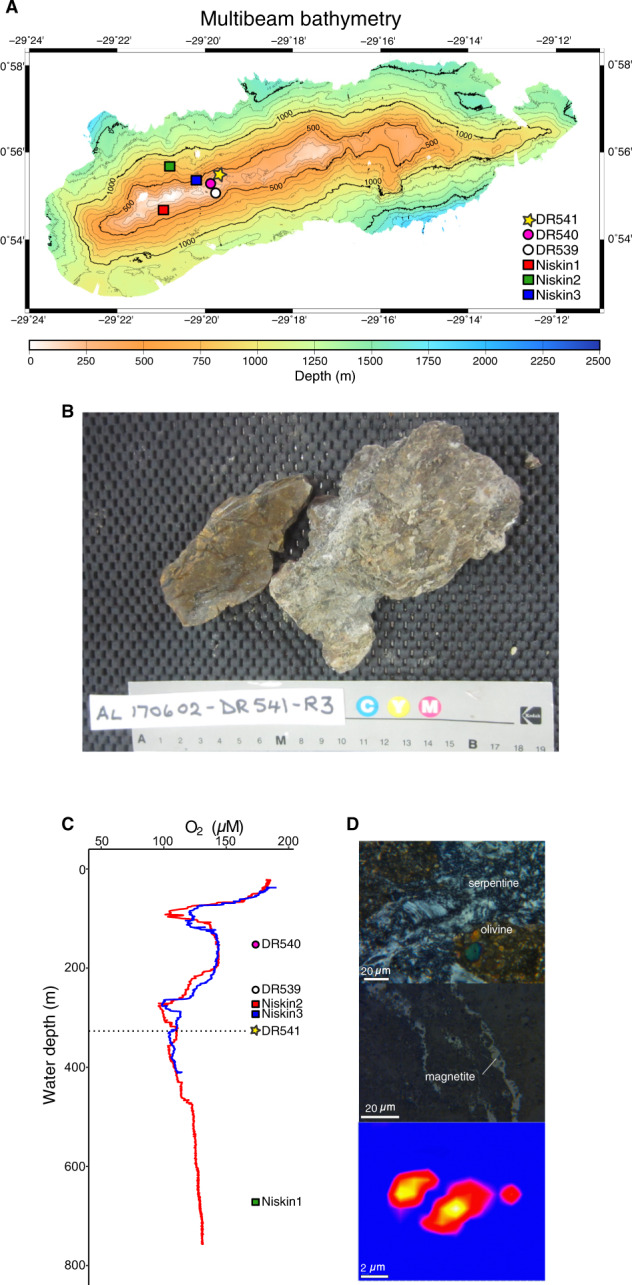
Bathymetry and petrology of SPSPA. **A** Bathymetry of SPSPA and sampling locations for Niskin, IGT fluids, sediments, and rocks. The location for the qSIP experiment (D541) is shown with a yellow star. **B** Photo of partially serpentinized peridotite collected from St. Pauls Rocks, the interior of the rock was used for the qSIP incubations. **C** Dissolved oxygen vertical profiles from two sites (Niskin2, Niskin3) in close proximity to the location for qSIP (D541). The horizontal dashed line represents the depth where samples were taken for qSIP (327 meters). The other labels indicate the water depths at which those samples were taken (see map in panel **A**). **D** Thin section photomicrographs of sheared peridotite from SPSPA. The presence of serpentine and magnetite (top two panels) is indicative of H_2_ generation. Hyperspectral Raman map (bottom panel) showing a CH_4_-rich inclusion in amphibole sampled from the close vicinity of the studied area.

Partially serpentinized peridotite (DR541-R3; 00°55.56′N; 29°19.70′W) and bottom seawater samples were collected from a yellowish-brown outcrop using the *Deep Rover* submersible in July 2017 at 327 m water depth from the northern slope east of the SPSPA, Brazil (M/V *Alucia* Expedition AL170602, 00°55′N; 29°21′W), a remote group of islets in the equatorial Atlantic Ocean, on the Mid-Atlantic ridge (Fig. 1A). The SPSPA belongs to the Brazilian Exclusive Economic Zone and is located within the Fernando de Noronha Environmental Protection Area. Conductivity, temperature, and density profiles were taken from several stations around the SPSPA to explore ongoing hydrothermal activity which could not be detected. The alteration mineralogy of serpentinized peridotite mylonite and fluid inclusion contents in primary minerals were determined in thin sections using a petrographic microscope and a confocal Raman spectrometer (Horiba LabRAM HR) equipped with a 20 mW 473 nm laser, astigmatic flat field spectrograph with a focal length of 800 mm, and a multichannel air-cooled (−70 °C) CCD detector.

Seawater was collected from directly above the peridotite rocks using an isobaric gas-tight (IGT) fluid sampler [18]. In addition, seawater was collected with a Niskin rosette from three nearby sites (Fig. 1A), two from 300 to 330 m water depth to serve as a background seawater microbial community comparison to the IGT fluids and peridotite rocks collected from dive DR541. Niskin (4–8 L) and IGT (18–75 mL) seawater for the *t*_0_ comparisons were filtered onto 0.2 µm polycarbonate filters using a peristaltic pump and frozen immediately at −20 °C.

Sediments were collected from two nearby sites (Fig. 1A) using the slurp suction sampler onboard the Deep Rover submersible. Sediments were stored in 50 mL falcon tubes at −20 °C until DNA extraction.

### DNA extraction

**Fig. 2:**
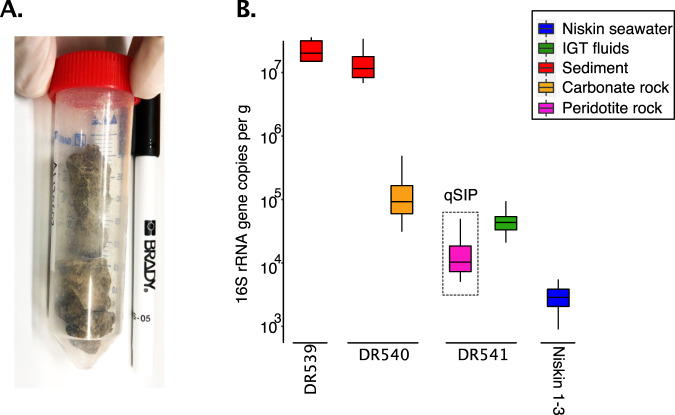
Microbial abundance in seafloor and seawater samples. **A** Photograph of peridotite rock samples (dive D541) prior to DNA extraction. **B** Concentration of 16S rRNA genes from sediments, rocks, IGT fluids, and Niskin collected seawater. Concentrations are normalized to per gram for sediment and rock samples, and per mL for seawater samples (IGT fluids and Niskins).

DNA was extracted from the seawater samples (frozen filters) using a protocol described previously [19]. In order to avoid cross-sample contamination with the rock samples, DNA was extracted from 10 to 12 g of rock samples on a separate day (Fig. 2A), in a laminar flow clean bench with pipettors that were autoclaved immediately before use (to remove contaminating DNA on the pipettors). DNA was extracted from three separate peridotite rock samples (subsamples of the same rock). These *t*_0_ rock samples were collected from the same dive (DR541-R3; Fig. 1B) where peridotite rocks were sampled for the qSIP incubations (Fig. 1A). In addition, two separate carbonate rock replicates (subsamples of the same rock) were collected from dive DR540 (DR540-R3 and R4) (Fig. 1A). All *t*_0_ rock samples were stored in 50 mL RNA/DNA clean falcon tubes (Fig. 2A), and DNA was extracted according to a previously published protocol [20]. The only deviation from the previous protocol was that silica glass beads from three Lysing Matrix E tubes (MP Biomedicals) were directly added to the 50 mL falcon tubes containing the rocks (Fig. 2A), which were homogenized with 10 mL C1 extraction buffer [20].

The *t*_0_ peridotite rocks (DR541-R3) serve as a reference for the in situ peridotite-associated microbial community and allow comparison against the “rock-free” community from the seawater samples. Moreover, because the *t*_0_ peridotite rocks (DR541-R3) were collected from the same location as the peridotites that were used for setting up the ^13^C-SIP incubations, we could identify “rock-associated” OTUs (detected in the peridotite *t*_0_ samples) that became labeled in the qSIP incubations that were, or were not, detected in seawater. For DNA extraction from sediments, we used the same protocol that we applied for the carbonate and peridotite rock samples, with the main difference that only 0.5 g of sediment was extracted in 2 mL lysing matrix E tubes with 1 mL of C1 extraction buffer [20]. Such a relatively small volume was required given the orders of magnitude higher microbial abundance in the sediment samples compared to the rocks (Fig. 2B).

### Experimental setup for SIP incubations

For incubation experiments, the outermost ~3 cm of rock sample DR541-R3 was carefully removed with a sterile hammer to retrieve the rock interior. The rock interior was subsequently crushed into mm-sized fragments on a sterile surface (ethanol washed) in a fume hood for incubations. For the inocula in the SIP incubations, 2 g of crushed rock fragments from the interior of the peridotites were placed into 20 mL gas-tight glass vials which had been heated to 450 °C for 10 h prior to use. In addition, 10 mL sterile-filtered seawater (using 0.2 µm polycarbonate filters) was added to each vial containing the crushed peridotite rocks. Therefore, the living cells in the SIP incubations should be primarily rock-associated and derived from the interior of the peridotites, as sterile filtration should have removed cells >0.2 µm in size from the added seawater. However, it is possible that some ultra-small seawater cells <0.2 µm could have passed through and made it into the SIP incubations.

Each vial was amended with either 10 mM sodium-[^13^C]bicarbonate (99% ^13^C-content, Sigma-Aldrich, Darmstadt, Germany), 2 mM sodium-[^13^C_1_]acetate or 10 mM sodium-[^13^C]formate (99% ^13^C-content, Cambridge Isotope Laboratories, Andover, MA, USA) and crimp-sealed using KOH-washed butyl stoppers [21]. We acknowledge that the high concentrations of ^13^C-acetate and ^13^C-formate that were added are orders of magnitude higher compared with the measured concentrations of acetate and formate in fluids venting from the Lost City Hydrothermal field [22]. All glass vials were crimp-sealed with a butyl rubber stopper creating gas-tight conditions and the atmosphere was replaced with nitrogen gas (N_2_) for 10 min to create low oxygen conditions. Afterward, one set of flasks was amended with H_2_ added to the headspace (1.5 bar), with a second set as a control that did not receive H_2_. We acknowledge that these concentrations are higher than those at most hydrothermal systems [7].

Although the O_2_ was not measured in the incubations, we assume dissolved oxygen concentration was reduced down ca. 10-fold compared to the ambient concentration of ca. 120 µM by purging the incubation medium for a minimum of 10 min with N_2_ [23]. The resulting low O_2_ conditions of assumed 10–15 µM were likely further drawn down by aerobic respiration during the course of the experiment [24].

As is common practice for all DNA-SIP studies, control vials were also prepared with the same unlabeled carbon sources (referred to as “unlabeled control”) to compare the extent of ^13^C-labeling from the labeled incubations. The glass vials containing unlabeled control and ^13^C-substrates were incubated at room temperature (ca. 25 °C) terminated after 35 h and stored at −60 °C for onshore analysis. DNA was extracted from 1 g of slurry in triplicate using the same method as described above [20] and quantified fluorometrically using Qubit 3.0 fluorometer (Invitrogen, Eugene, OR, USA).

### Density gradient centrifugation and gradient fraction

DNA samples were prepared for density gradient centrifugation according to previously defined protocols for qSIP [13, 14]. DNA of density fractions was resuspended with 30 µl molecular-grade (DEPC-treated) water and quantified fluorometrically using a Qubit fluorometer.

### Quantitative PCR (qPCR)

Universal primers targeting the V4 hypervariable region of 16S ribosomal RNA (rRNA) genes were used in qPCR to determine density shifts in the peak DNA of buoyant density (BD) for each incubation. We used a version of the 515F primer with a single-base change (in bold) to increase the coverage of archaeal groups (515F-Y, 5′-GTG**Y**CAGCMGCCGCGGTAA [25]). qPCR was carried out as described previously [26]. 16S rRNA gene quantities of the density fractions were plotted against their corresponding densities and 10 fractions (on average) from each replicate set were selected for sequencing (Fig. S1; gray shaded area). Two 16S rRNA gene PCR amplicons from each density fraction (technical replicates to reduce PCR bias) were pooled and subjected to dual-indexed barcoded sequencing of 16S rRNA gene amplicons on the MiniSeq (Illumina) as described previously [27].

### Bioinformatic and qSIP analysis

The MiniSeq reads were quality trimmed and assembled using USEARCH version 11.0.667 with the default parameters [28] resulting in 6.8 million quality checked V4 reads. Reads were then de novo clustered at 97% identity using UPARSE; OTUs represented by a single sequence were discarded [29]. Taxonomic assignments were generated by QIIME 1.9.1 [30] using the implemented BLAST method against the SILVA rRNA gene database release 132 [31]. The raw OTU table consisted of 10,654 OTUs which were further quality-filtered. The level of contamination in each density fraction for qSIP analysis was determined using previously sequenced DNA sequences from dust samples collected from three different laboratories in our building where the samples are processed [27]. If the total number reads per OTU in the samples was 10 times greater compared to that of contaminant sequence reads, the OTU was considered to be endemic to the sample. 1662 contaminant-related OTUs such as *Pseudomonas*, *Ralstonia*, *Variovorax*, or *Streptococcus* [32] were deleted based on comparing contaminant sample sequence reads to the samples, comprising 2.34% of the whole 16S rRNA gene dataset (188,273 sequence reads out of 8,046,165). Only OTUs having >12 sequences in total in each replicate (summed across all density fractions) were selected for further study since low abundance taxa cause artificial variations in qSIP calculations [33]. Intermediate files in data removal of contaminants, quality filtering, and detailed explanations of the intermediate files can be found in the following data repository: 10.6084/m9.figshare.13341443.v1.

Excess atomic fraction ^13^C (EAF) values were calculated for the 16S rRNA genes corresponding to OTUs according to a previously described study [13] using a qSIP workflow embedded in the HTS-SIP R package [34]. To calculate the bootstrap confidence intervals (CI) for significant isotope incorporation, bootstrap replicates (*n* = 1000) were run with the HTS-SIP R package; an OTU was considered as a ^13^C-assimilator if the lower boundary of CI was above the 0% EAF cutoff [13]. Statistical analyses and plots were performed using R.Studio Version 3.3.0 [35].

qSIP measurements of OTU-specific ^13^C-substrate assimilation with and without H_2_ allowed us to test whether the activity of microbial communities in the presence of H_2_ was significantly restricted to specific phylogenetic clades (e.g., “phylogenetic signal” [33]). Pagel’s *λ* and Blomberg’s *K* were calculated as two independent indices of the phylogenetic signal [36, 37]: shared traits (e.g., ^13^C-assimilation patterns) in the context of evolutionary history (e.g., 16S rRNA gene phylogenetic relation).

### Metagenomic analysis of rock, seawater, and SIP samples

Given the shifts in buoyant density of 16S rRNA genes in the ^13^C-SIP incubations (Fig. S1), we produced metagenomes from “heavy” fractions of the density gradients that indicated ^13^C labeling (Fig. S1). The DNA contained within the density fraction for each of these “heavy” metagenomes was chosen for metagenomic shotgun sequencing based on the region of the CsCl gradient that exhibited a peak in the ^13^C-substrate incubation that had a higher CsCl density compared to the unlabeled control experiment (Fig. S1). Metagenomic libraries were prepared using Nextera XT DNA Library Prep Kit (Illumina) and following the manual provided by the manufacturer with minor modifications. The starting concentration of genomic DNA could not be set to 0.2 ng as suggested by the manufacturer’s manual due to low DNA content in the labeled SIP fractions. Instead, the PCR program in the amplification step of the fragmented DNA was increased from 12 to 15 cycles. Metagenomic libraries from the rock and seawater *t*_0_ samples were prepared from the extracted DNA (see above) with unique barcodes using the same Nextera XT kit, were diluted to 1 nM, and pooled together for sequencing on the MiniSeq (Illumina) platform.

Paired-end reads were trimmed and assembled into contigs using CLC Genomics Workbench 9.5.4 (Qiagen, Hilden, Germany), using a word size of 20, bubble size of 50, and a minimum contig length of 300 nucleotides. Reads were then mapped to the contigs using the following parameters (mismatch penalty, 3; insertion penalty, 3; deletion penalty, 3; minimum alignment length, 50% of reading length; minimum percent identity, 95%). Coverage values were obtained from the number of reads mapped to a contig divided by its length (i.e., average coverage). This protocol does not assemble rRNA genes [38]; thus, results are only discussed in terms of protein-encoding genes.

For annotating putative functions of ORFs in metagenomes from particular “higher-level” taxonomic groups of microorganisms, we applied a previously published bioinformatics pipeline [38]. This pipeline extracts protein-encoding ORFs from de novo-assembled contigs using FragGeneScan v. 1.30 [39], and functionally annotates ORFs against a large aggregated database (“MetaProt”) [38] using DIAMOND version 0.9.24 [40]. The MetaProt database contained predicted proteins from all protist, fungal, bacterial, and archaeal genomes (and MAGs) in the JGI and NCBI databases as of January 2021. The MetaProt database [38] also contains ORFs from all of the transcriptomes of microbial eukaryotes from the MMETS project [41]. The MetaProt database is available as a single 32 GB amino acid fasta file on the LMU Open Data website (https://data.ub.uni-muenchen.de/183/). Cutoff values for assigning hits to specific taxa were performed at a minimum bit score of 50, the minimum amino acid similarity of 60, and an alignment length of 50 residues. All scripts and code used to produce the analysis have been posted on GitHub (https://github.com/williamorsi/MetaProt-database). This approach assigns ORFs to higher-level taxonomic groups [38]. As is the case in all metagenomic studies, the incomplete nature of genomes in databases, together with the lower representation of sequenced genomes from candidate clades than from cultured ones, makes it likely that our pipeline misses annotation of ORFs that are derived from as-yet-unsequenced genomes.

The 16S rRNA gene amplicon sequences and metagenomic sequence data were entered in the NCBI Sequence Read Archive under BioProject ID PRJNA679196. The CTD data, metagenomic dataset, and intermediate files to produce qSIP results were deposited under https://figshare.com/authors/_mer_Coskun/9725927.

### Assessing biases in metagenomes from density fractions containing ^13^C-enriched DNA

Sequencing metagenomic DNA from only a single ^13^C-enriched fraction (Fig. S1) may be biased due to [1] low GC genomes that might not be detected in ^13^C-enriched fraction even though they highly incorporate the labeled source, and [2] abundant organisms can sometimes be found in all fractions irrespectively of labeling [42, 43]. However, many abundant ^13^C-labeled OTUs were determined with statistical significance via qSIP related to *Marinobacter, Alteromonas*, Thaumarchaeota, ‘*Ca*. Rokubacteria’, or Nitrospinae, were represented in the ^13^C-metagenomes. The overlapping taxa labeled in qSIP and metagenomes from ^13^C-enriched SIP fractions indicate that the metagenomic sequences obtained from the selected SIP fractions are derived to a large extent from taxa that were ^13^C-labeled.

### Phylogenetic analyses

For phylogenetic analyses of ^13^C-labeled 16S rRNA genes, OTUs which were at least occurring in one of the experiments were selected for alignment with MUSCLE [44] using SeaView [45]. The resulting fasta file was imported into W-IQ-TREE [46] with an option to select the best phylogenetic model using Bayesian criterion, which resulted in TIM3e + R10 algorithm using ModelFinder [47]. The phylogenetic tree was visualized and edited using iTOL [48]. Statistical analyses and plots were performed using R.Studio Version 3.3.0 [35]. Pagel’s *λ* [37] and Blomberg’s *K* [36] tests for significantly non-random phylogenetic distributions of ^13^C-utiliziers from qSIP were calculated using the phylosignal R package [49].

For phylogenetic analyses of ORFs from metagenomes with similarity to *HypE*, *nirS*, and *coxL* based on BLASTp searches against the MetaProt database [38], ORFs were aligned against their top BLASTp hits using MUSCLE [44]. Phylogenetic analysis of the resulting amino acid alignments of the predicted proteins was conducted in SeaView using RAxML [50] with BLOSUM62 as the evolutionary model and 100 bootstrap replicates. The resulting phylogenetic trees were displayed as unrooted cladograms using FigTree (http://tree.bio.ed.ac.uk/software/figtree/).

## Results

### Rock description

Thin section petrography and Raman analysis revealed that rock sample DR541-R3 (used as the inoculum for the qSIP incubations) is a partially serpentinized peridotite mylonite that is chiefly composed of olivine and orthopyroxene, minor amounts of clinopyroxene, and traces of Cr-spinel (Fig. 1D). Primary minerals are partially altered to serpentine, magnetite, tremolite, calcite, and aragonite which chiefly occur in veins cutting across the mylonite matrix. We observed methane-rich fluid inclusions at other locations at SPSPA but not at the sampling location (Fig. 1D). For comparison, we obtained thin sections of two additional samples from the same dive (samples DR541-R1 and DR541-R4) which revealed structural and alteration patterns similar to those of DR541-R3 suggesting all three samples experienced extensive ductile and brittle deformation followed by serpentinization.

### Microbial abundance and diversity of the ultramafic-rock associated community

**Fig. 3:**
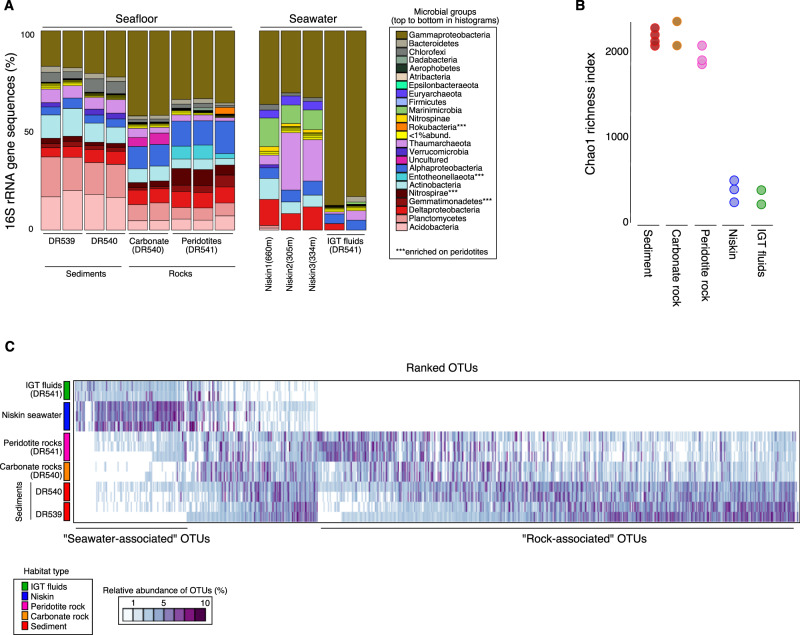
Microbial diversity in seafloor and seawater samples. **A** The taxonomic composition of sediment, rock, and seawater communities. **B** Chao1 estimated microbial richness, showing a significantly higher richness in the rock and sediments samples, compared to the seawater communities (two-sided *T*-test: *P* = e^−8^). **C** Heatmap showing the relative abundance of OTUs (columns) per sample (rows) and their distribution across sample types. The community detected on the ultramafic rocks was significantly different compared to the seawater communities (ANOSIM: *P* = 0.001). More than half of the total OTUs detected were found only in the seafloor samples (rocks and sediments).

The concentration of 16S rRNA genes from the peridotite rock samples was 2.6 (±2)×10^4^ copies per g of rock, compared to 7 (±1)×10^4^ and 0.5 (±0.06)×10^4^ 16S rRNA gene copies per mL seawater collected with the IGT fluid samplers and Niskin rosette, respectively (Fig. 2B). qPCR quantification of 16S rRNA gene copies from the frozen peridotite rock samples showed cycle threshold (*C*_t_) values ranging between 25 and 30 cycles, which strongly indicates that our amplified 16S rRNA genes are derived from in situ microbes associated with the rocks as opposed to contamination because all contamination controls (extraction blanks and qPCR no template controls) consistently had *C*_t_ values >35. The Chao diversity index based on the 16S rRNA gene data shows that the rock-associated community is significantly more diverse (two-sided *T*-test: *P* < 0.0001) compared to the seawater communities (Fig. 3B). 16S rRNA gene data also shows that the microbial community composition between the peridotite-associated and the seawater communities was significantly different (Analysis of Similarity [ANOSIM] *R*: 0.87, *P* < 0.0001), with approximately two-thirds of all detected OTUs being found on the peridotite rock samples and not in any seawater samples (Fig. 3C). The peridotite-associated communities included taxa that are common to hydrothermal [51] and ultramafic rock habitats [52, 53] including Nitrospirae, Rokubacteria, Entotheonellaeota, Gemmatimonadetes, and Alphaproteobacteria (Fig. 3A). These comparisons reveal a unique microbial community inhabiting the peridotite rocks compared to the seawater that was collected directly above these rocks with the IGT fluid samplers.

#### Identifying ^13^C-labeling for qSIP

^13^C-labeling of 16S rRNA genes (defined by a shift in peak DNA buoyant density) was observed in all incubations, with average shifts in the peak ^13^C-DNA buoyant densities compared to control-incubations between 0.0018 ± 0.0037 and 0.0279 ± 0.0076 g ml^−1^ (Fig. S1). This highlights biological variability in ^13^C-assimilation in our replicate treatments, but despite this variability, the average density shift in the replicate treatments shows a clear trend of ^13^C-enriched DNA from 16S rRNA genes compared to the unlabeled controls (Fig. S1). Therefore, ^13^C-labeling of microbes synthesizing new DNA occurred in all incubations. The use of replicates in qSIP allows for statistically constrained estimations of ^13^C-assimilation in all detectable OTUs [13]. Namely, ^13^C-qSIP allows for the calculation of the ^13^C-excess atomic fraction (EAF) for all detectable OTUs within a microbial community, together with a confidence interval (CI) that provides to test statistical significance for OTU-specific ^13^C-assimilation [13]. We thus applied the qSIP protocol to estimate the ^13^C-EAF (with CI) for all detected OTUs within each experimental treatment.

#### Significance of H_2_ on the phylogenetic organization of ^13^C-utilizing taxa

Phylogenetic signal analyses showed that H_2_ had a statistically significant (non-random) effect on the phylogenetic organization of the ^13^C-assimilating taxa in each of the three substrates tested (Table 1). Thus, the effect of H_2_ on increased ^13^C-labeling of clades with ^13^C-bicarbonate and ^13^C-acetate, and to a lesser extent ^13^C-formate, was statistically significant in terms of the non-random distribution of specific taxa that increased their anabolism with added H_2_.

**Table 1:** The result of phylogenetic signal tests (Blomberg’s *K* and Pagel’s *λ*) analysis on the effect of H_2_ on carbon assimilation.

	*K*	*λ*	Labeled OTUs	Unlabeled OTUs	Labeled OTUs (%)
Bicarbonate	0.13	0	81	58	58.3
Bicarbonate + H_2_	0.26*	0.64	26	61	29.9
Acetate	0.11	0	73	79	48
Acetate + H_2_	0.25*	0.82	81	17	82.7
Formate	0.16	0	59	6	90.8
Formate + H_2_	0.25*	0.64	37	129	22.3
Total number of OTUs	–	–	107	203	34.5

*p ≤ 0.05.

**Fig. 4:**
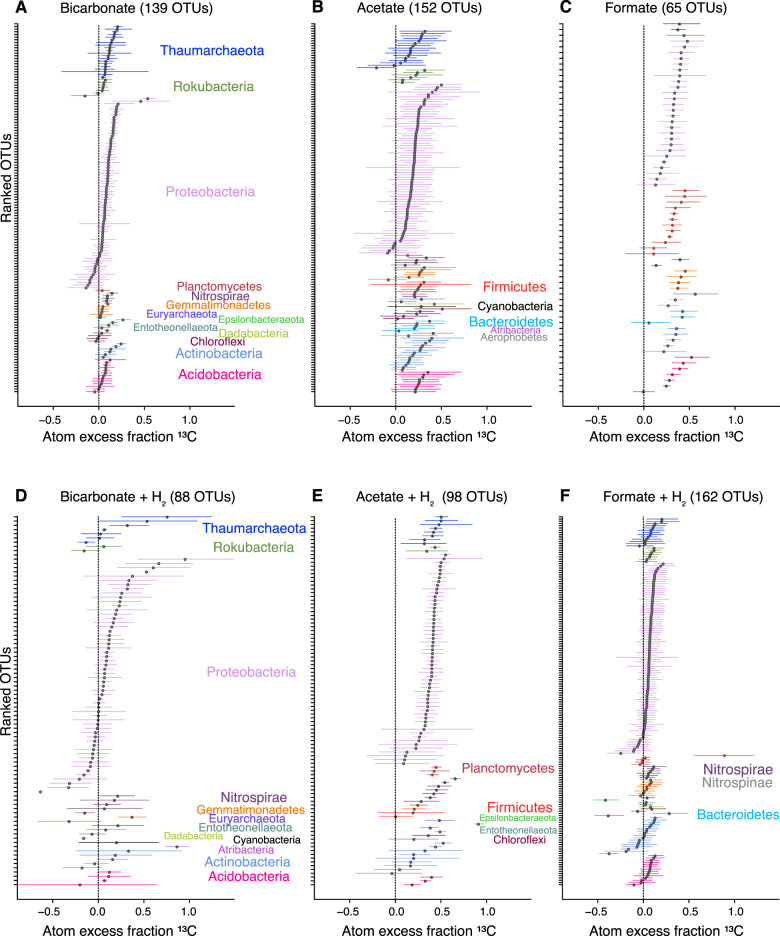
OTU-specific shifts in the median atom fraction excess (^13^C) of OTUs with 90% confidence interval (CI). Individual points represent EAF values of specific OTUs, which are colored by Phylum for qSIP incubations that did not (**A**–**C**), or did (**D**–**F**), receive H_2_. The error bars correspond to 90% CI across three biological replicates. OTUs that do not have a 90% CI overlapping with 0 are considered to be ^13^C labeled.

**Fig. 5:**
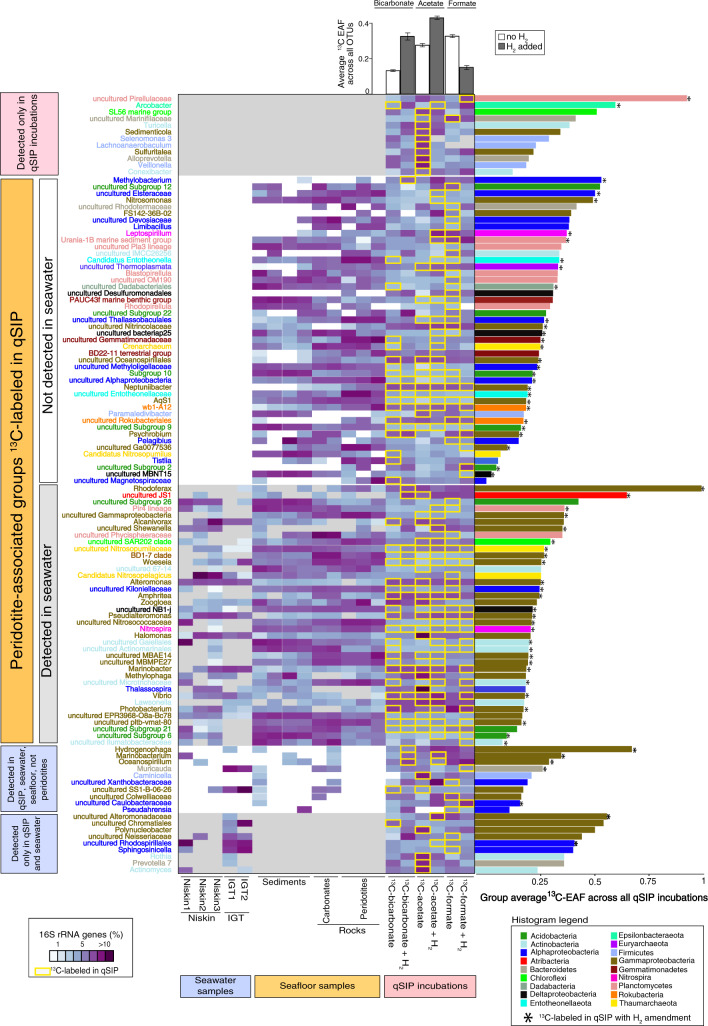
The distribution of carbon assimilating groups within the rock, sediment, and seawater samples. The heatmap shows the relative abundance of 16S rRNA gene sequences per group per sample (purple = more, light blue = less). Within the qSIP incubations (right-hand side), groups that had OTUs with statistically significant ^13^C-assimilation in qSIP are surrounded by a yellow box. The histograms on the right side of the plot show the average ^13^C-EAF across all qSIP incubations per group. An asterisk indicates that this group had a significant ^13^C-EAF within a qSIP incubation with added H_2_ (no asterisk means labeling occurred only in qSIP incubations without H_2_). The histograms at the top of the heatmap show the average ^13^C-EAF across all OTUs within each qSIP incubation, error bars represent standard deviations. The analysis shows that the majority of ^13^C-assimilating groups were rock-associated and that more rock-associated groups became labeled in the presence of H_2_ (middle of the heatmap), compared to the seawater-associated groups (at the bottom of the heatmap).

With added H_2_, OTUs increased assimilation of ^13^C-bicarbonate and ^13^C-acetate on average by 146% (±29%) and 55% (±34%), respectively (Fig. 4). Moreover, within the peridotite rock-associated OTUs, 54 genus-level taxonomic groups were ^13^C-labeled in qSIP incubations containing added H_2_ (Fig. 5). This was more than an order of magnitude higher compared to seawater-specific ^13^C-labeled taxa, as well as seafloor-associated ^13^C-labeled taxa not detected on peridotite rocks (only detected on carbonate rocks and sediments) (Fig. 5).

#### The effects of H_2_ on bicarbonate assimilation

Compared to controls (^13^C-bicarbonate incubations that did not receive H_2_) H_2_ addition was correlated with an overall increase in ^13^C-assimilation by OTUs with EAF values increasing on average from 0.13 ± 0.009 to 0.32 ± 0.051 (146 ± 29% increase) (Fig. 4). Most of these OTUs were rock-associated (Fig. 5). H_2_ addition coincided with a two-fold drop in ^13^C-labeled Gammaproteobacterial OTUs (Fig. 4). In contrast, the abundance and ^13^C-bicarbonate assimilation (EAF) of Thaumarchaeal OTUs in the presence of H_2_ increased to 7.79% and 0.38 ± 0.34, respectively (Fig. 4), which were associated with the peridotite rocks (Fig. 5). H_2_ addition coincided with a ^13^C-bicarbonate assimilating peridotite-associated OTU affiliated with the Atribacteria (JS1 clade) having the highest EAF of all OTUs in the ^13^C-bicarbonate incubations amended with H_2_ (EAF: 0.87) (Fig. 4). In total, 85% of the ^13^C-bicarbonate assimilating taxa were associated with the peridotite rocks (Fig. 5).

#### Effects of H_2_ on acetate assimilation

In ^13^C-acetate incubations with added H_2_, the EAF value per OTU increased from 0.27 ± 0.010 to 0.42 ± 0.011 (55 ± 34% increase) relative to control incubations that did not receive H_2_ (Fig. 4). Gammaproteobacteria and Thaumarchaeota had the highest number of OTUs that increased ^13^C-acetate assimilation in the presence of H_2_ (Fig. 4). The genera showing the highest ^13^C-acetate assimilation in the presence of H_2_ were *Arcobacter* (0.91 EAF), *Marinobacterium* (0.54 EAF), *Nitrosomonas* (0.49 EAF), *Alteromonas* (0.42 EAF), Nitrosopumilaceae (0.4 EAF), and *Nitrospira* (0.14 EAF) (Fig. 4). All of the acetate assimilating Nitrosopumilaceae (Thaumarchea) and Nitrospira taxa were peridotite-associated (Fig. 5).

#### Effects of H_2_ on formate assimilation

In contrast to acetate and bicarbonate incubations, OTUs assimilating ^13^C-formate in the presence of H_2_ had on average lower EAF values compared to the control ^13^C-formate qSIP incubations that did not receive H_2_ (Figs. 4 and 5) (average 0.32 ± 0.01–0.14 ± 0.02 EAF; 139 ± 11% decrease). This shows that the utilization of formate by most microbial groups was reduced in the presence of H_2_. The composition of the community in the H_2_-supplemented formate incubation changed substantially, with OTUs belonging to the Planctomycetes (Family Pirellulaceae) and Entotheonellaeota exhibiting the highest EAF values from ^13^C-formate (up to 0.89 EAF) (Figs. 4 and 5). All of the Planctomycetes and Entotheonellaeota formate assimilating taxa were associated with the peridotite rocks (Fig. 5).

#### Functional gene diversity in peridotite-associated clades assimilating ^13^C-substrates

**Fig. 6:**
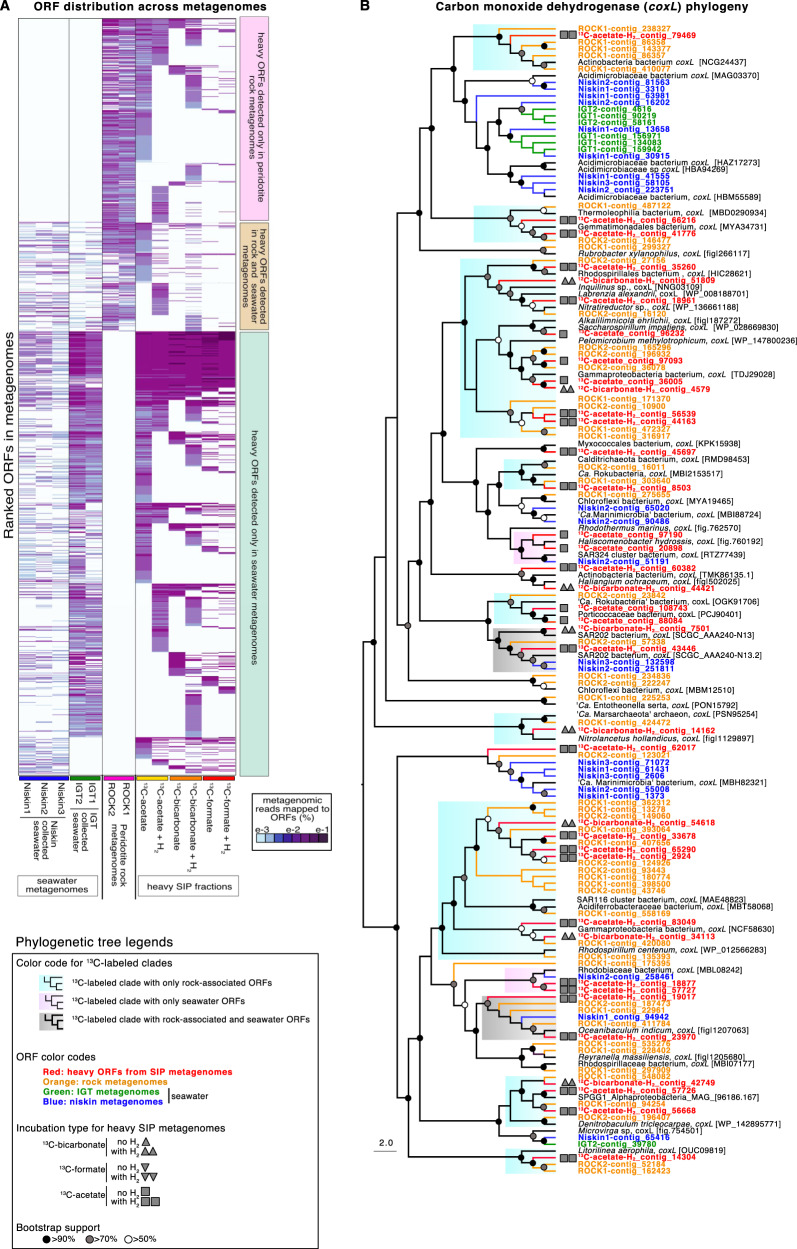
Metagenome ORF distribution and *coxL* phylogeny. **A** Heatmap displaying all ORFs detected from heavy SIP metagenomes and the distribution of these heavy ORFs in the rock and seawater metagenomes. Heatmap rows represent predicted proteins from the MetaProt database [38] having best BLASTp similarity to peptides encoded in ORFs from the metagenomes (columns). Colors represent length normalized read coverage from metagenomes to the ORFs. **B** Phylogenetic analysis (RAxML) of all detected *coxL* ORFs based on an alignment length of 968 amino acids. Note that most of the *coxL* clades were rock-specific and that these also contain the majority of heavy *coxL* ORFs.

**Fig. 7:**
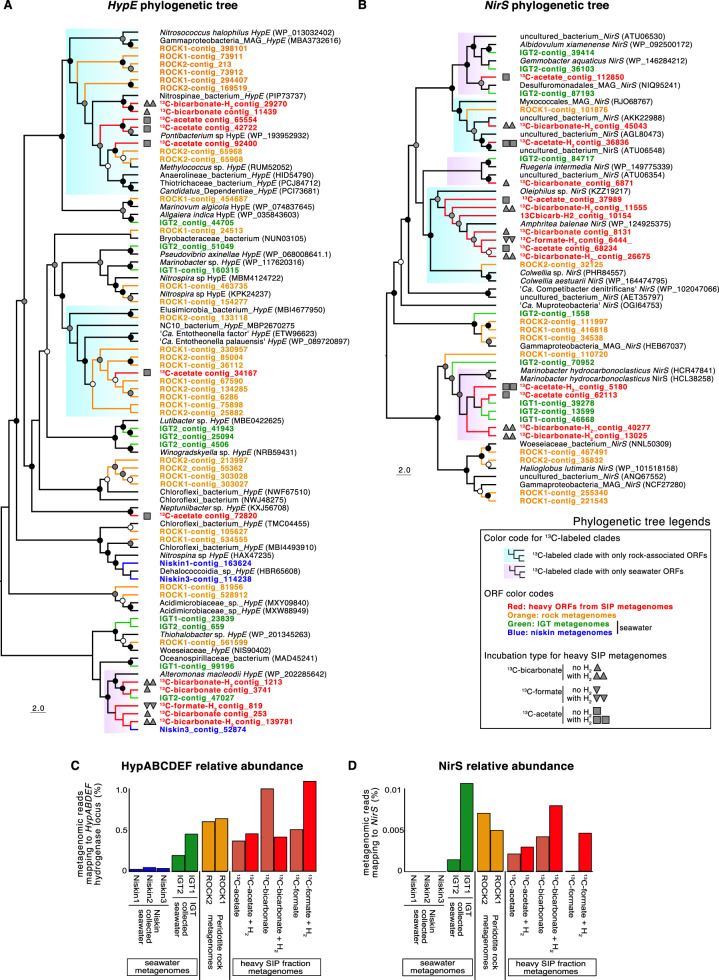
Distribution of *HypE* and *nirS* in metagenomes. Phylogenetic analyses (RAxML) of all detected *HypE* (**A**) and *nirS* (**B**) ORFs based on alignment lengths of 442 and 638 amino acids, respectively. Note that the separation of seawater (pink highlighted) and rock-associated (light blue highlighted) clades. Panels (**C**) and (**D**) show the relative abundance (% reads mapping, length normalized) within the different metagenomes to ORFs with best BLASTp similarity to the NiFe-hydrogenase assembly locus *HypABCDEF* (**C**) and *nirS* (**D**), respectively. Circles on nodes represent bootstrap values (black > 90%, gray > 70%, white > 50%).

In the heavy metagenomes, approximately one-third of ORFs were associated with peridotite rocks and not with seawater (Fig. 6A). This is evidence of a rock-associated community with unique protein-encoding gene content that was assimilating ^13^C. Within the heavy metagenomes, diversity of carbon monoxide dehydrogenase (*coxL*: Fig. 6B), H_2_-oxidizing NiFe-hydrogenase assembly proteins (*HypE*: Fig. 7A), and dissimilatory nitrite reductase (*nirS*: Fig. 7B) all show bootstrap supported clades that contained ORFs from the heavy metagenomes and peridotite metagenomes. The majority (>75%) of these clades did not include any ORFs from seawater metagenomes (Figs. 6 and 7). This indicates that most of the *coxL*, *HypE* and *nirS* encoding organisms that assimilated the ^13^C-substrates in the SIP incubations were associated primarily with the peridotites.

In the peridotite rock metagenomes, there was an increased relative abundance of ORFs encoding *nirS* and NiFe-hydrogenase assembly proteins involved in H_2_ oxidation (*HypABCDEF*) [54] compared to Niskins and IGT collected water, which was consistent across biological replicates (Fig. 7C and D). Relative abundance of *HypABCDEF* and *nirS* encoding ORFs in heavy metagenomes from SIP-incubations were also higher compared to seawater metagenomes (Fig. 7C and D).

Phylogenetic analysis of the carbon monoxide dehydrogenase large subunit (*coxL*) encoding ORFs reveals nine major bootstrap-supported *coxL* clades that include ORFs from heavy metagenomes and peridotite rock metagenomes but did not contain any *coxL* ORFs from seawater metagenomes (Fig. 6B). The peridotite-associated *coxL* clades contain a four-fold higher number (6 compared to 25) of heavy ORFs compared to seawater-associated *coxL* clades, and were affiliated with *Labrenzia*, *Pelomicrobium*, *Denitrobaculum*, *Nitrolancetus*, *Litorilnea*, uncultivated Actinobacteria, SAR116 clade, and ‘*Ca*. Rokubacteria’ (Fig. 6B).

## Discussion

The increased diversity of the peridotite-associated community compared to seawater microbial communities (Fig. 3B) is consistent with prior studies of seafloor communities associated with basaltic rocks [55]. The majority of taxa that assimilated ^13^C in the presence of H_2_ were derived from this diverse peridotite-associated community as opposed to being specific to seawater (Fig. 5). Therefore, the peridotite-associated communities are enriched with the capability to utilize H_2_ to increase their carbon assimilation from CO_2_, acetate, and formate. Since H_2_, acetate, and formate are formed during serpentinization [56], our results highlight the importance of H_2_ in influencing carbon cycling in rock-hosted microbial communities.

The presence of magnetite in the serpentinized rock matrix suggests that temperatures exceeded 200 °C when rocks underwent serpentinization [57]. Because its formation requires the oxidation of ferrous iron originally contained in primary minerals to ferric iron in magnetite with water as the oxidizing agent, H_2_ was generated during serpentinization of mylonite at St. Paul’s Rocks. Because the mylonite was only partially serpentinized, it is likely that low-temperature aqueous alteration at SPSPA is currently ongoing—albeit at slow rates—which would be a source of H_2_ for the peridotite-hosted communities. Unlike peridotite-hosted alkaline hydrothermal vents with a focused flow such as the Atlantis Massif [58], we found no firm evidence of H_2_ anomalies in the water column at SPSPA indicating that any H_2_ had already been oxidized in the water column that has ca. 100 µM O_2_ (Fig. 1C). However, H_2_ in diffuse fluids emanating from the subseafloor of SPSPA could be oxidized by the rock-associated seafloor microbes (Figs. 3C, 5) before diffusing into the oxidized water column, as indicated previously by geochemical evidence in diffuse vents from the Juan de Fuca Ridge [59].

### Carbon assimilation in an ultramafic rock-associated community

The observation that most ^13^C-assimilating taxa were peridotite-associated (Fig. 5), indicates that many taxa within this relatively diverse community (Fig. 3B) have a high affinity for the added substrates compared to the seawater microbes living directly above the rocks. Gammaproteobacteria dominate the ^13^C-labeled taxa that were detected in seawater, but the ^13^C-assimilating taxa detected only in the seafloor samples were by comparison more diverse and instead consist of Thaumarchaea, Rokubacteria, Planctomycetes, Acidobacteria, Entotheonellaeota, Deltaproteobacteria, and Gemmatimonadaceae (Fig. 5). The qSIP results show that the effect of H_2_ on increased carbon assimilation is phylogenetically organized in this unique rock-associated community, with specific peridotite-associated taxa assimilating more carbon in the presence of H_2_ compared to seawater-associated taxa (Fig. 5).

The phylogenetic signal analysis based on Blomberg’s *K* [36] reveals H_2_ utilization as a shared trait in ^13^C-assimilating taxa, compared to the incubations that did not receive H_2_ (Table 1, Fig. S2). This significant phylogenetic signal within ^13^C-assimilating taxa in the presence of added H_2_ coincides with an order of magnitude higher number of peridotite rock associated taxa having an increased anabolism in the presence of added H_2_, compared to taxa not detected on the ultramafic rocks (Fig. 5). These relations point to ongoing H_2_ production via low-temperature aqueous alteration of peridotite (Fig. 1D) which supports a unique peridotite-associated community with higher diversity (Fig. 3B) that is relatively enriched with the ability to metabolize H_2_ compared to the overlying seawater communities (Fig. 7C).

#### Differential effects of H_2_ on the assimilation of acetate, bicarbonate, and formate

The increased ^13^C-assimilation with added H_2_ (Figs. 4 and 5) is supported by a higher relative abundance of the *HypABCDEF* [NiFe]-hydrogenase locus in heavy metagenomes from H_2_-amended incubations (Fig. 7C). The *HypABCDEF* locus is responsible for the maturation of NiFe-hydrogenase in bacteria and aerobic H_2_ oxidation [54]. The *HypE* ORFs within clades exhibiting ^13^C assimilation were affiliated with uncultured Nitrospinae bacteria, *Nitrosococcus*, ‘*Ca*. Entotheonella’, and *Alteromonas* (Fig. 7A), which were all groups identified in qSIP as being rock-associated with increased carbon assimilation in the presence of H_2_ (Fig. 5). The higher carbon assimilation in the presence of added H_2_ in several rock-associated groups (Fig. 5) are consistent with H_2_ oxidation via *HypABCDEF*, and could be related to higher H_2_ concentrations in the peridotite ecosystem.

The correlation of added H_2_ with a decreased formate assimilation by most OTUs (Fig. 4) is possibly due to inhibition of the hydrogenase unit of formate hydrogen lyase that is caused by high H_2_ concentrations [60]. This has been observed in previous SIP studies, where H_2_ had an inhibitory effect on formate assimilation in hot springs [10]. However, microcosm experiments with H_2_ and formate in terrestrial alkaline fluids in the Samail Ophiolite showed that under high H_2_ concentrations (20%, excess atmospheric pressure) certain methanogens increase methane production from formate, presumably via formate dehydrogenase [61]. Therefore, in serpentinization settings the inhibition or stimulation of formate metabolism by increased H_2_ concentrations is likely to be dependent on the redox potential of the environment.

### Atribacteria exhibit H_2_-dependent carbon fixation

Of the all OTUs that had enhanced carbon assimilation in H_2_-amended ^13^C-bicarbonate incubations, a single rock-associated OTU affiliated with Atribacteria had the highest EAF value (0.87; Fig. 4). This fits with the presence of an H_2_-dependent CO_2_ fixation pathway (Wood–Ljungdahl pathway) encoded in Atribacteria genomes [62, 63]. The labeling of the Atribacteria OTU with ^13^C-bicarbonate was not observed in the absence of H_2_ (Fig. 4), indicating that H_2_ is needed to fix bicarbonate by this taxon.

The same rock-associated Atribacteria OTU also incorporated ^13^C-acetate, but only in the absence of H_2_ (Fig. 4). However, the amount of ^13^C-acetate assimilated by this OTU was ca. 50% less compared to its assimilation of ^13^C-bicarbonate in the presence of H_2_. These results possibly reflect the reversibility of the Wood–Ljungdahl pathway encoded in the Atribacteria genomes [62, 63]. For example, when H_2_ is present Atribacteria use the Wood–Ljungdahl pathway in the forward direction to fix CO_2_ and produce either acetyl-CoA or acetate [64]. However, when H_2_ is not present the activity of acetate kinase may be reversed and acetate is assimilated [64]. In line with our finding, it has been recently discussed that Atribacteria could use WLP either in catabolic or anabolic directions in deep subseafloor sediments [65].

Atribacteria typically dominates CH_4_-rich anoxic environments such as gas hydrate-containing sediments, and methanogenic meromictic lakes [66]. Since our sampled environment was oxic at the time of sampling (Fig. 1C), it seems that some Atribacteria is able to persist under these conditions and increase their carbon fixation activity upon the addition of H_2_.

### Effects of H_2_ on carbon assimilation by nitrogen-cycling Bacteria and Archaea

OTUs belonging to the ammonia-oxidizing Thaumarchaeota (Nitrosopumilaceae) had a significant increase in EAF values in the presence of added H_2_ in the ^13^C-bicarbonate and ^13^C-acetate incubations (Fig. 4). All of these OTUs were peridotite-associated (Fig. 5). To our knowledge, there are no experimental studies with pure cultures demonstrating that Thaumarchaea catabolize H_2_. However, the genomes of some ammonia-oxidizing archaea (AOA) encode NiFe hydrogenase genes that may potentially be involved in H_2_ oxidation [67–70] which might explain the increased ^13^C-assimilation of Thaumarchaea in the presence of H_2_ seen here.

The increased assimilation of ^13^C-bicarbonate and ^13^C-acetate by rock-associated Thaumarchaeal OTUs in H_2_ amended incubations with (Figs. 4, 5) indicates many of the AOA used H_2_ to increase their mixotrophic activity. Mixotrophy by AOA is a well-known feature [71, 72], and our data indicate it can be increased in the presence of H_2_. However, some AOA have higher ammonia oxidation rates at lower oxygen concentrations [73], and the H_2_ amended incubations should have promoted lower oxygen conditions. It is thus possible that higher activity in some rock-associated Thaumarchaeota was further stimulated by reduced oxygen concentrations. Additional experiments are required to determine whether the increased ^13^C assimilation of mixotrophic AOA seen here is due to H_2_ oxidation, low oxygen, or a combination of both.

Nitrospirae are nitrite-oxidizing bacteria (NOB) and were found to be enriched in peridotite rock samples (Figs. 3 and 5) and also exhibited a ^13^C-labeling pattern consistent with a H_2_ catabolism. Namely, *Nitrospira*-affiliated OTUs increased their assimilation of acetate in the H_2_-amended incubations by 64.6% (±36%) (Figs. 4 and 5). This is in line with H_2_ oxidation demonstrated for NOB in pure culture experiments [54].

The higher relative abundance of *nirS* encoding ORFs in peridotite rock and IGT metagenomes compared to the Niskin seawater metagenomes (Fig. 7D) could be explained by low-oxygen levels commonly experienced at the benthic-seawater interface selecting for anaerobic, nitrite respiring bacteria [74]. The relative abundance of heavy *nirS* ORFs was higher in the presence of added H_2_ (Fig. 7D), which indicates that the H_2_ was selected for nitrite respiring bacteria. Most of the heavy *nirS* ORFs were affiliated with *Marinobacter* and *Amphritea* (Fig. 7B), which rock-associated and exhibited some of the highest levels of ^13^C-assimilation with added H_2_ (Figs. 4 and 5). Taken together, these results indicate that the addition of H_2_ promoted increased assimilation of ^13^C via anaerobic nitrate-reducing bacteria, including peridotite-associated *Marinobacter* and *Amphritea*.

The addition of H_2_ affected increased carbon assimilation of peridotite-associated ammonia and nitrite-oxidizing consortia (Fig. 5) that are responsible for nitrification. Together with the activity of rock-associated nitrite reducing bacteria (Fig. 7), our results show the potential for H_2_ to effect coupled nitrification and denitrification in the rock-associated community. Coupled nitrification and denitrification influence the loss of fixed nitrogen from benthic ecosystems [75]. Our findings raise the possibility that serpentinization derived H_2_ may influence fixed nitrogen loss (as N_2_ gas) from the peridotite-rock associated ecosystem, by stimulating the activity of nitrifiers and denitrifiers. Nitrogen cycling consortia have been found in terrestrial serpentinization systems as well, such as the Somali Ophiolite system in Oman [76]. The possibility for abiotically produced H_2_ from serpentinization reactions to influence fixed nitrogen loss via coupled nitrification and denitrification in ultramafic rock ecosystems is a topic worthy of future study.

### Carbon assimilation by CO oxidizers in the rock-associated community

Carbon monoxide can serve as the sole source of carbon and energy for life in environments that are low in an organic matter [77], including terrestrial serpentinization settings [78, 79]. The *coxMSL* enzyme (carbon monoxide dehydrogenase) catalyzes the oxidation of CO (carbon monoxide) to CO_2_ in bacteria [80], and is a widespread mechanism supporting microbial survival [81, 82], particularly in extreme habitats experiencing low levels of productivity [4]. The bootstrap-supported separation of seawater and rock-associated *coxL* clades indicate a unique rock-associated community capable of using CO as an energy source. The substrate utilization within these peridotite-rock-associated clades of *coxL* encoding organisms appears to have been affected by H_2_ amendments. For example, heavy *coxL* ORFs from SIP incubations amended with H_2_ were detected within bootstrap-supported peridotite-associated clades affiliated with the ‘*Ca*. Rokubacteria’, Gemmatimonadales, and SAR202 clade (Fig. 6B). This raises the possibility that CO oxidation might be related to anabolism at relatively high H_2_ concentrations by some taxa within these groups.

The carbon assimilation by CO-oxidizing bacteria associated with ultramafic rocks seen here could be explained by the water–gas shift reaction (CO_2_ + H_2_ = CO + H_2_O) [83]. Our data shows that in seafloor ultramafic rock settings where O_2_ is above detection and H_2_ is likely produced via serpentinization or related low-temperature alteration processes, aerobic CO-oxidizing organisms become stimulated by CO that is produced via the abiotic reduction of CO_2_ with H_2_. All of the CO-dehydrogenases that we detected were the molybdenum–copper-containing form which functions in aerobic CO-oxidation to CO_2_, as opposed to the NiFe CO-dehydrogenases that function in anaerobic CO_2_ reduction to CO [83]. Our results from peridotite-associated communities at SPSPA are similar to terrestrial serpentinization settings, where aerobic CO oxidation supports life that survives under alkaline conditions [78, 79].

Taken together, the phylogenetic analysis of *coxL*, *HypE*, and *nirS* show a similar pattern: the majority of heavy ORFs cluster together in bootstrap supported clades of peridotite rock-associated taxa. This trend suggests that the oxidation of H_2_ and carbon monoxide, as well as dissimilatory nitrite reduction, are widespread and important physiological features for taxa that were assimilating ^13^C within the peridotite-associated microbial community.

### Assessing effects of the qSIP incubation conditions

A comparison of effects of the substrates (acetate, formate, bicarbonate) is problematic due to the extraordinarily high concentrations of acetate and formate added relative to the in situ conditions, and potential substrate inhibition, toxicity, and pH alterations. Therefore, we do not compare the effects of the substrates to one another (e.g., we do not claim that bicarbonate is a more important carbon source compared to formate, despite the clear differences in qSIP results) but rather compare assimilation of a particular substrate with, and without, added H_2_. While the added concentrations in our incubations are higher than the in situ abundances (no H_2_ above background was detected in the water column), our experimental approach provides initial boundary conditions on the stimulation of carbon assimilation by H_2_ for specific substrates, by specific peridotite-associated taxa in an ultramafic seafloor setting undergoing low-temperature aqueous alteration.

An increased temperature of the incubations (room temperature) relative to the in situ temperature (10–12 °C), probably led to elevated microbial activity and rates of ^13^C substrate assimilation as shown previously for benthic microbes [84]. Moreover, purging of the incubation flasks with N_2_ created dissolved O_2_ concentrations at low oxygen levels (see the “Methods” section), and the labeling of known strictly anaerobic taxa indicates that anoxic regions were established during the incubation. Because O_2_ was available at low concentrations in the flasks (ca. 10 µM), this explains why many of the most highly ^13^C-enriched taxa in the qSIP are known aerobic or facultatively anaerobic taxa (Figs. 4 and 5). However, at the flask bottom below the 3 cm column of crushed peridotite rock, anoxic conditions likely established due to a vertical O_2_ gradient in the flask that commonly occurs in this experimental setup due to increased rates of aerobic respiration at the benthic–water interface [24]. Anoxic conditions in the crushed rocks at the bottom of the flasks likely promoted carbon assimilation by rock-associated strict anaerobes that also were using H_2_ to increase their anabolism from the added ^13^C-labeled substrates. For example, this is seen in the Atribacteria that are strict anaerobes [85] that exhibited the highest ^13^C-bicarbonate assimilation in the presence of H_2_ (Fig. 4). Moreover, the ^13^C-labeling of a peridotite-associated taxon affiliated with the Firmicute *Paramaledivibacter* (Fig. 5) indicates anoxic conditions, as this is a strictly anaerobic organism originally isolated from a deep-sea hydrothermal vent from the Mid-Atlantic Ridge [86].

### Assessing cross-feeding of ^13^C-labeled substrates

Cross feeding is an issue inherent to all SIP studies. It is possible that some of the ^13^C was fixed from bicarbonate into organic molecules and subsequently assimilated by heterotrophs. The labeling of heterotrophic taxa with ^13^C-bicarbonate indicates that some of the ^13^C-bicarbonate was taken up by autotrophs and assimilated by heterotrophs as DOM or POM. Alternatively, heterotrophic carbon fixation occurs through anaplerotic carbon fixation reactions in heterotrophs that can account for 2–8% of cell carbon [87, 88] and could explain a portion of the ^13^C labeling in heterotrophs seen here. It is not possible that the ^13^C from the organic substrates would be remineralized and be taken up as bicarbonate because the natural bicarbonate concentration in seawater is ~2.3 mM and will dilute the remineralized ^13^C label to undetectable levels [19]. Therefore, the results in bicarbonate incubations not only show the primary utilizers but underpin complex ecological interactions in the microbial food web. We also note that formate can rapidly interconvert with CO_2_ and therefore some of the formate may have been taken up as ^13^C-bicarbonate [89]. These results support the hypothesis that anaplerotic carbon fixation reactions in heterotrophs represent an important, yet underappreciated, component of the global marine carbon cycle [90].

## Conclusions

**Fig. 8:**
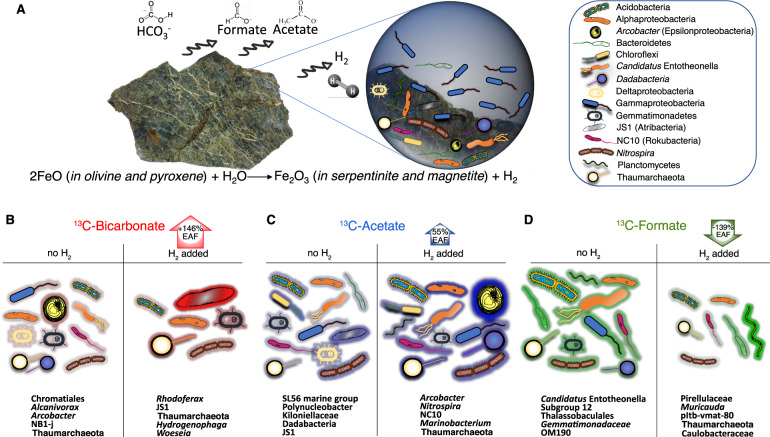
Summary of group-specific qSIP-labeling patterns. **A** The photograph is a piece of rock from partially serpentinized peridotite that was taken from SPSPA for the qSIP incubations. The cartoon diagram in the upper right shows the corresponding groups that assimilated carbon in the presence (or absence) of H_2_. **B**–**D** The plots below show the main groups that were responsible for carbon assimilation of different substrates with, and without H_2_. The extent of red/blue/green shading on the outside of the cells corresponds to ^13^C-labeling from CO_2_/acetate/formate. The percentage number (inside the arrows) indicates the average increase or decrease in ^13^C-assimilation (EAF) in the community with the addition of H_2_.

Our findings demonstrate that H_2_ has a quantitatively significant impact on microbial carbon assimilation in seafloor ultramafic rock microbial ecosystems (Fig. 8). The qSIP results show that this effect of H_2_ on increased carbon assimilation is phylogenetically organized, and the distribution of the carbon assimilating taxa shows a higher diversity of peridotite-associated taxa assimilating carbon in the presence of H_2_ compared to taxa that were not detected on the ultramafic rocks. The data support thermodynamic predictions that oxidation of H_2_ is energetically favorable for seafloor-associated microbial life in settings where H_2_-containing aqueous solutions mix with seawater [7], including those where H_2_ is formed via high-temperature or low-temperature aqueous alteration [91, 92], rock comminution in fault zones, and radiolysis.

## Supplementary information

Supplemental Materials

## References

[1] Schink B (1997). Energetics of syntrophic cooperation in methanogenic degradation. Microbiol Mol Biol Rev.

[2] Vignais PM, Billoud B (2007). Occurrence, classification, and biological function of hydrogenases: an overview. Chem Rev.

[3] Wolf PG, Biswas A, Morales SE, Greening C, Gaskins HR (2016). H_2_ metabolism is widespread and diverse among human colonic microbes. Gut Microbes.

[4] Ji M, Greening C, Vanwonterghem I, Carere CR, Bay SK, Steen JA (2017). Atmospheric trace gases support primary production in Antarctic desert surface soil. Nature.

[5] Islam ZF, Welsh C, Bayly K, Grinter R, Southam G, Gagen EJ (2020). A widely distributed hydrogenase oxidises atmospheric H_2_ during bacterial growth. ISME J.

[6] Greening C, Biswas A, Carere CR, Jackson CJ, Taylor MC, Stott MB (2016). Genomic and metagenomic surveys of hydrogenase distribution indicate H_2_ is a widely utilised energy source for microbial growth and survival. ISME J.

[7] Amend JP, McCollom TM, Hentscher M, Bach W (2011). Catabolic and anabolic energy for chemolithoautotrophs in deep-sea hydrothermal systems hosted in different rock types. Geochim Cosmochim Acta.

[8] Reveillaud J, Reddington E, McDermott J, Algar C, Meyer JL, Sylva S (2016). Subseafloor microbial communities in hydrogen-rich vent fluids from hydrothermal systems along the Mid-Cayman Rise. Environ Microbiol.

[9] Perner M, Hansen M, Seifert R, Strauss H, Koschinsky A, Petersen S (2013). Linking geology, fluid chemistry, and microbial activity of basalt- and ultramafic-hosted deep-sea hydrothermal vent environments. Geobiology.

[10] Schubotz F, Hays LE, Meyer-Dombard D, Gillespie A, Shock EL, Summons RE (2015). Stable isotope labeling confirms mixotrophic nature of streamer biofilm communities at alkaline hot springs. Front Microbiol.

[11] Fortunato CS, Huber JA (2016). Coupled RNA-SIP and metatranscriptomics of active chemolithoautotrophic communities at a deep-sea hydrothermal vent. ISME J.

[12] McNichol J, Stryhanyuk H, Sylva SP, Thomas F, Musat N, Seewald JS (2018). Primary productivity below the seafloor at deep-sea hot springs. Proc Natl Acad Sci USA.

[13] Hungate BA, Mau RL, Schwartz E, Caporaso JG, Dijkstra P, van Gestel N (2015). Quantitative microbial ecology through stable isotope probing. Appl Environ Microbiol.

[14] Coskun ÖK, Pichler M, Vargas S, Gilder S, Orsi WD (2018). Linking uncultivated microbial populations with benthic carbon turnover using quantitative stable isotope probing. Appl Environ Microbiol.

[15] Tuorto SJ, Darias P, McGuinness LR, Panikov N, Zhang T, Häggblom MM (2014). Bacterial genome replication at subzero temperatures in permafrost. ISME J.

[16] Maia M, Sichel S, Briais A, Brunelli D, Ligi M, Ferreira N (2016). Extreme mantle uplift and exhumation along a transpressive transform fault. Nat Geosci.

[17] Klein F, Tarnas JD, Bach W (2020). Abiotic sources of molecular hydrogen on Earth. Elements.

[18] Seewald JS, Doherty KW, Hammar TR, Liberatore SP (2002). A new gas-tight isobaric sampler for hydrothermal fluids. Deep Sea Res Part I.

[19] Orsi WD, Smith JM, Liu S, Liu Z, Sakamoto CM, Wilken S (2016). Diverse, uncultivated bacteria and archaea underlying the cycling of dissolved protein in the ocean. ISME J.

[20] Vuillemin A, Wankel SD, Coskun OK, Magritsch T, Vargas S, Estes ER (2019). Archaea dominate oxic subseafloor communities over multimillion-year time scales. Sci Adv.

[21] Oremland RS, Miller LG, Whiticar MJ (1987). Sources and flux of natural gases from Mono Lake, California. Geochim Cosmochim Acta.

[22] Lang SQ, Butterfield DA, Schulte M, Kelley DS, Lilley MD (2010). Elevated concentrations of formate, acetate and dissolved organic carbon found at the Lost City hydrothermal field. Geochim Cosmochim Acta.

[23] Butler IB, Schoonen MA, Rickard DT (1994). Removal of dissolved oxygen from water: a comparison of four common techniques. Talanta.

[24] Ortega-Arbulu AS, Pichler M, Vuillemin A, Orsi WD (2019). Effects of organic matter and low oxygen on the mycobenthos in a coastal lagoon. Environ Microbiol.

[25] Parada AE, Needham DM, Fuhrman JA (2016). Every base matters: assessing small subunit rRNA primers for marine microbiomes with mock communities, time series and global field samples. Environ Microbiol.

[26] Coskun ÖK, Özen V, SD Wankel SD, Orsi WD. Quantifying population-specific growth in benthic bacterial communities under low oxygen using H_2_^18^O. ISME J. 2019;13:1546–59. 10.1038/s41396-019-0373-4 PMC6776007 30783213

[27] Pichler M, Coskun ÖK, Ortega-Arbulú A-S, Conci N, Wörheide G, Vargas S, et al. A 16S rRNA gene sequencing and analysis protocol for the Illumina MiniSeq platform. Microbiologyopen 2018:7;e00611. 10.1002/mbo3.611 PMC6291791 29575567

[28] Edgar RC (2010). Search and clustering orders of magnitude faster than BLAST. Bioinformatics.

[29] Edgar RC (2013). UPARSE: highly accurate OTU sequences from microbial amplicon reads. Nat Methods.

[30] Caporaso JG, Kuczynski J, Stombaugh J, Bittinger K, Bushman FD, Costello EK (2010). QIIME allows analysis of high-throughput community sequencing data. Nat Methods.

[31] Quast C, Pruesse E, Yilmaz P, Gerken J, Schweer T, Yarza P (2013). The SILVA ribosomal RNA gene database project: improved data processing and web-based tools. Nucleic Acids Res.

[32] Salter SJ, Cox MJ, Turek EM, Calus ST, Cookson WO, Moffatt MF (2014). Reagent and laboratory contamination can critically impact sequence-based microbiome analyses. BMC Biol.

[33] Morrissey EM, Mau RL, Schwartz E, Caporaso JG, Dijkstra P, van Gestel N (2016). Phylogenetic organization of bacterial activity. ISME J.

[34] Youngblut ND, Barnett SE, Buckley DH (2018). HTSSIP: an R package for analysis of high throughput sequencing data from nucleic acid stable isotope probing (SIP) experiments. PLoS ONE.

[35] R. Team. (2015). Others, RStudio: integrated development for R. vol. 42.

[36] Blomberg SP, Garland T, Ives AR (2003). Testing for phylogenetic signal in comparative data: behavioral traits are more labile. Evolution.

[37] Pagel M (1999). Inferring the historical patterns of biological evolution. Nature.

[38] Orsi WD, Morard R, Vuillemin A, Eitel M, Worheide G, Milucka J (2020). Anaerobic metabolism of Foraminifera thriving below the seafloor. ISME J.

[39] Rho M, Tang H, Ye Y (2010). FragGeneScan: predicting genes in short and error-prone reads. Nucleic Acids Res.

[40] Buchfink B, Xie C, Huson DH (2015). Fast and sensitive protein alignment using DIAMOND. Nat Methods.

[41] Keeling PJ, Burki F, Wilcox HM, Allam B, Allen EE, Amaral-Zettler LA (2014). The Marine Microbial Eukaryote Transcriptome Sequencing Project (MMETSP): illuminating the functional diversity of eukaryotic life in the oceans through transcriptome sequencing. PLoS Biol.

[42] Sieradzki ET, Koch BJ, Greenlon A, Sachdeva R, Malmstrom RR, Mau RL (2020). Measurement error and resolution in quantitative stable isotope probing: implications for experimental design. mSystems.

[43] Youngblut ND, Barnett SE, Buckley DH (2018). SIPSim: a modeling toolkit to predict accuracy and aid design of DNA-SIP experiments. Front Microbiol.

[44] Edgar RC (2004). MUSCLE: multiple sequence alignment with high accuracy and high throughput. Nucleic Acids Res.

[45] Gouy M, Guindon S, Gascuel O (2010). SeaView version 4: a multiplatform graphical user interface for sequence alignment and phylogenetic tree building. Mol Biol Evol.

[46] Trifinopoulos J, Nguyen L-T, von Haeseler A, Minh BQ (2016). W-IQ-TREE: a fast online phylogenetic tool for maximum likelihood analysis. Nucleic Acids Res.

[47] Kalyaanamoorthy S, Minh BQ, Wong TKF, von Haeseler A, Jermiin LS (2017). ModelFinder: fast model selection for accurate phylogenetic estimates. Nat Methods.

[48] Letunic I, Bork P (2016). Interactive tree of life (iTOL) v3: an online tool for the display and annotation of phylogenetic and other trees. Nucleic Acids Res.

[49] Keck F, Rimet F, Bouchez A, Franc A (2016). phylosignal: an R package to measure, test, and explore the phylogenetic signal. Ecol Evol.

[50] Stamatakis A (2006). RAxML-VI-HPC: maximum likelihood-based phylogenetic analyses with thousands of taxa and mixed models. Bioinformatics.

[51] Meier DV, Pjevac P, Bach W, Markert S, Schweder T, Jamieson J (2019). Microbial metal-sulfide oxidation in inactive hydrothermal vent chimneys suggested by metagenomic and metaproteomic analyses. Environ Microbiol.

[52] Lecoeuvre A, Menez B, Cannat M, Chavagnac V, Gerard E (2021). Microbial ecology of the newly discovered serpentinite-hosted Old City hydrothermal field (southwest Indian ridge). ISME J.

[53] Mason OU, Di Meo-Savoie CA, Van Nostrand JD, Zhou J, Fisk MR, Giovannoni SJ (2009). Prokaryotic diversity, distribution, and insights into their role in biogeochemical cycling in marine basalts. ISME J.

[54] Koch H, Galushko A, Albertsen M, Schintlmeister A, Gruber-Dorninger C, Lucker S (2014). Growth of nitrite-oxidizing bacteria by aerobic hydrogen oxidation. Science.

[55] Santelli CM, Orcutt BN, Banning E, Bach W, Moyer CL, Sogin ML (2008). Abundance and diversity of microbial life in ocean crust. Nature.

[56] Schrenk MO, Brazelton WJ, Lang SQ (2013). Serpentinization, carbon, and deep life. Rev Mineral Geochem.

[57] Klein F, Bach W, Humphris SE, Kahl W-A, Jöns N, Moskowitz B (2014). Magnetite in seafloor serpentinite—some like it hot. Geology.

[58] Kelley DS, Karson JA, Früh-Green GL, Yoerger DR, Shank TM, Butterfield DA (2005). A serpentinite-hosted ecosystem: the Lost City hydrothermal field. Science.

[59] Wankel SD, Germanovich LN, Lilley MD, Genc G, DiPerna CJ, Bradley AS (2011). Influence of subsurface biosphere on geochemical fluxes from diffuse hydrothermal fluids. Nat Geosci.

[60] McDowall JS, Murphy BJ, Haumann M, Palmer T, Armstrong FA, Sargent F (2014). Bacterial formate hydrogenlyase complex. Proc Natl Acad Sci USA.

[61] Fones EM, Colman DR, Kraus EA, Stepanauskas R, Templeton AS, Spear JR (2021). Diversification of methanogens into hyperalkaline serpentinizing environments through adaptations to minimize oxidant limitation. ISME J.

[62] Carr SA, Orcutt BN, Mandernack KW, Spear JR (2015). Abundant Atribacteria in deep marine sediment from the Adélie Basin, Antarctica. Front Microbiol.

[63] Nobu MK, Dodsworth JA, Murugapiran SK, Rinke C, Gies EA, Webster G (2016). Phylogeny and physiology of candidate phylum ‘Atribacteria’ (OP9/JS1) inferred from cultivation-independent genomics. ISME J.

[64] Schuchmann K, Müller V (2016). Energetics and application of heterotrophy in acetogenic bacteria. Appl Environ Microbiol.

[65] Vuillemin A, Vargas S, Coskun OK, Pockalny R, Murray RW, Smith DC (2020). Atribacteria reproducing over millions of years in the Atlantic Abyssal subseafloor. mBio.

[66] Rinke C, Schwientek P, Sczyrba A, Ivanova NN, Anderson IJ, Cheng JF (2013). Insights into the phylogeny and coding potential of microbial dark matter. Nature.

[67] Bryant FO, Adams MW (1989). Characterization of hydrogenase from the hyperthermophilic archaebacterium, *Pyrococcus furiosus*. J Biol Chem.

[68] Berney M, Greening C, Conrad R, Jacobs WR, Cook GM (2014). An obligately aerobic soil bacterium activates fermentative hydrogen production to survive reductive stress during hypoxia. Proc Natl Acad Sci USA.

[69] Kwan P, McIntosh CL, Jennings DP, Hopkins RC, Chandrayan SK, Wu C-H (2015). The [NiFe]-hydrogenase of *Pyrococcus furiosus* exhibits a new type of oxygen tolerance. J Am Chem Soc.

[70] Daebeler A, Herbold CW, Vierheilig J, Sedlacek CJ, Pjevac P, Albertsen M (2018). Cultivation and genomic analysis of “*Candidatus* Nitrosocaldus islandicus,” an obligately thermophilic, ammonia-oxidizing Thaumarchaeon from a hot spring biofilm in Graendalur Valley, Iceland. Front Microbiol.

[71] W Qin W, Amin SA, Martens-Habbena W, Walker CB, Urakawa H, Devol AH (2014). Marine ammonia-oxidizing archaeal isolates display obligate mixotrophy and wide ecotypic variation. Proc Natl Acad Sci USA.

[72] Seyler LM, McGuinness LR, Gilbert JA, Biddle JF, Gong D, Kerkhof LJ. Discerning autotrophy, mixotrophy and heterotrophy in marine TACK archaea from the North Atlantic. FEMS Microbiol Ecol 2018;94:fiy014. 10.1093/femsec/fiy014 29390107

[73] Bristow LA, Dalsgaard T, Tiano L, Mills DB, Bertagnolli AD, Wright JJ (2016). Ammonium and nitrite oxidation at nanomolar oxygen concentrations in oxygen minimum zone waters. Proc Natl Acad Sci USA.

[74] Diaz R, Rosenberg R (1995). Marine benthic hypoxia: a review of its ecological effects and the behavioural response of benthic macrofauna. Oceanogr Mar Biol.

[75] Jenkins MC, Kemp WM (1984). The coupling of nitrification and denitrification in two estuarine sediments. Limnol Oceanogr.

[76] Rempfert KR, Miller HM, Bompard N, Nothaft D, Matter JM, Kelemen P (2017). Geological and geochemical controls on subsurface microbial life in the Samail Ophiolite, Oman. Front Microbiol.

[77] Ragsdale SW (2004). Life with carbon monoxide. Crit Rev Biochem Mol Biol.

[78] Fones EM, Colman DR, Kraus EA, Nothaft DB, Poudel S, Rempfert KR (2019). Physiological adaptations to serpentinization in the Samail Ophiolite, Oman. ISME J.

[79] Morrill PL, Brazelton WJ, Kohl L, Rietze A, Miles SM, Kavanagh H (2014). Investigations of potential microbial methanogenic and carbon monoxide utilization pathways in ultra-basic reducing springs associated with present-day continental serpentinization: the Tablelands, NL, CAN. Front Microbiol.

[80] Wilcoxen J, Zhang B, Hille R (2011). Reaction of the molybdenum- and copper-containing carbon monoxide dehydrogenase from Oligotropha carboxydovorans with quinones. Biochemistry.

[81] Cordero PRF, Bayly K, Man Leung P, Huang C, Islam ZF, Schittenhelm RB (2019). Atmospheric carbon monoxide oxidation is a widespread mechanism supporting microbial survival. ISME J.

[82] Seewald JS, Zolotov MY, McCollom T (2006). Experimental investigation of single carbon compounds under hydrothermal conditions. Geochim Cosmochim Acta.

[83] Can M, Armstrong FA, Ragsdale SW (2014). Structure, function, and mechanism of the nickel metalloenzymes, CO dehydrogenase, and acetyl-CoA synthase. Chem Rev.

[84] Gudasz C, Bastviken D, Steger K, Premke K, Sobek S, Tranvik LJ (2010). Temperature-controlled organic carbon mineralization in lake sediments. Nature.

[85] Katayama T, Nobu MK, Kusada H, Meng XY, Hosogi N, Uematsu K (2020). Isolation of a member of the candidate phylum ‘Atribacteria’ reveals a unique cell membrane structure. Nat Commun.

[86] Brisbarre N, Fardeau M-L, Cueff V, Cayol J-L, Barbier G, Cilia V (2003). *Clostridium caminithermale* sp. nov., a slightly halophilic and moderately thermophilic bacterium isolated from an Atlantic deep-sea hydrothermal chimney. Int J Syst Evol Microbiol.

[87] Roslev P, Larsen MB, Jørgensen D, Hesselsoe M (2004). Use of heterotrophic CO_2_ assimilation as a measure of metabolic activity in planktonic and sessile bacteria. J Microbiol Methods.

[88] Spona-Friedl M, Braun A, Huber C, Eisenreich W, Griebler C, Kappler A, et al. Substrate-dependent CO_2_ fixation in heterotrophic bacteria revealed by stable isotope labelling. FEMS Microbiol Ecol 2020;96:fiaa080. 10.1093/femsec/fiaa080 32358961

[89] Jansen K, Thauer RK, Widdel F, Fuchs G (1984). Carbon assimilation pathways in sulfate reducing bacteria. Formate, carbon dioxide, carbon monoxide, and acetate assimilation by *Desulfovibrio baarsii*. Arch Microbiol.

[90] Braun A, Spona-Friedl M, Avramov M, Elsner M, Baltar F, Reinthaler T, et al. Reviews and syntheses: heterotrophic fixation of inorganic carbon—significant but invisible flux in global carbon cycling. Biogeosciences 2020;18:3689–3700.

[91] Russell MJ, Hall AJ, Martin W. Serpentinization as a source of energy at the origin of life. Geobiology. 2010;8:355–71. 10.1111/j.1472-4669.2010.00249.x 10.1111/j.1472-4669.2010.00249.x 20572872

[92] Martin W, Baross J, Kelley D, Russell MJ. Hydrothermal vents and the origin of life. Nat Rev Microbiol. 2008;6:805–14. 10.1038/nrmicro1991. 10.1038/nrmicro1991 18820700

