# Correction to: Quantifying the effects of hydrogen on carbon assimilation in a seafloor microbial community associated with ultramafic rocks

**DOI:** 10.1038/s41396-021-01087-6

**Published:** 2021-11-30

**Authors:** Ömer K. Coskun, Aurèle Vuillemin, Florence Schubotz, Frieder Klein, Susanna E. Sichel, Wolfgang Eisenreich, William D. Orsi

**Affiliations:** 1 Department of Earth and Environmental Sciences, Ludwig-Maximilians-Universität, Munich, Germany; 2 MARUM Center for Marine Environmental Sciences, University of Bremen, Bremen, Germany; 3 Woods Hole Oceanographic Institution, Woods Hole, MA USA; 4 Departamento de Geologia e Geofísica/LAGEMAR–Universidade Federal Fluminense-Brazil, Niterói, RJ Brazil; 5 Department of Chemistry, Bavarian NMR Center–Structural Membrane Biochemistry, Technische Universität München, Garching, Germany; 6 GeoBio-CenterLMU, Ludwig-Maximilians-Universität München, Munich, Germany; 7 Present Address: GFZ German Research Centre for Geosciences, Helmholtz Centre Potsdam, Potsdam, Germany

**Keywords:** Microbial ecology, Water microbiology, Microbial biooceanography, Microbiome, Biogeochemistry

Correction to: *The ISME Journal* 10.1038/s41396-021-01066-x, published online 26 July 2021

The original version of this article unfortunately contained mistakes in references 85–91.

The original article has been corrected.

