# Supplementary material for: Quantifying the effects of hydrogen on carbon assimilation in a seafloor microbial community associated with ultramafic rocks
Source: ISME J. 2021 Jul 26;16(1):257–71. doi: 10.1038/s41396-021-01066-x (PMC8692406; doi:10.1038/s41396-021-01066-x)

**SUPPLEMANTARY FIGURES**


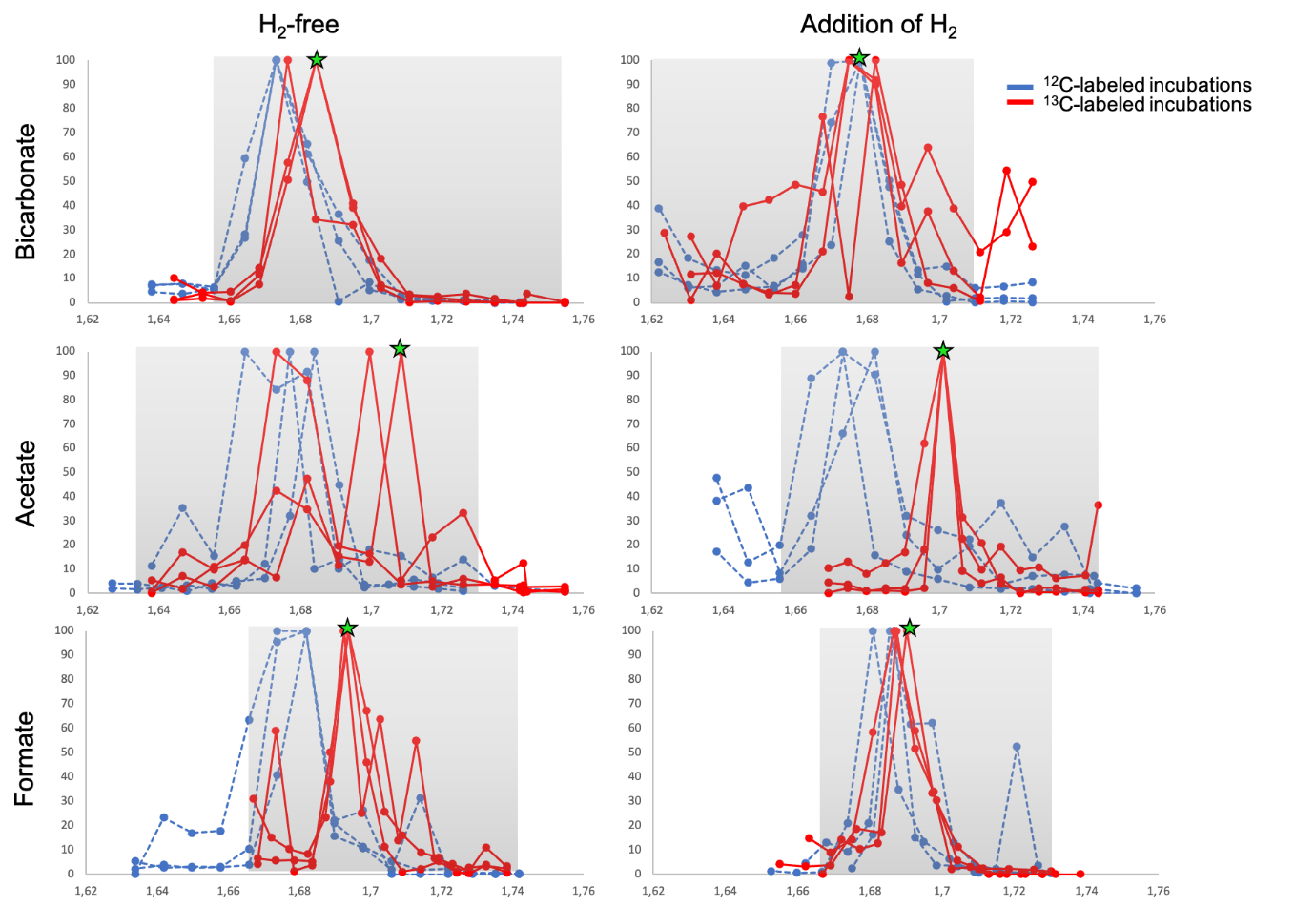


**Figure S1:** Quantification of 16S rRNA gene copies across CsCl density gradient fractions fractionated from incubations amended with bicarbonate, acetate and formate in the presence or absence of molecular H_2_. ^13^C-substrates are represented by red solid lines and unlabeled replicates (control) are represented by blue dashed lines. The y axis represents the relative abundance of 16S rRNA genes quantified with qPCR, normalized to maximal abundance across all density fractions. The green stars show the fractions that were used for metagenomic library preparation.

**Figure S2**: **Phylogeny of bacterial and archaeal 16S rRNA genes and their ^13^C-labeling with and without hydrogen.** The heat blocks correspond to excess atom fraction (EAF) of ^13^C-labeled OTUs and represent the following incubations from inner circle to outer: bicarbonate, bicarbonate amended with H_2_, acetate, acetate amended with H_2_, formate and formate amended with H_2_.


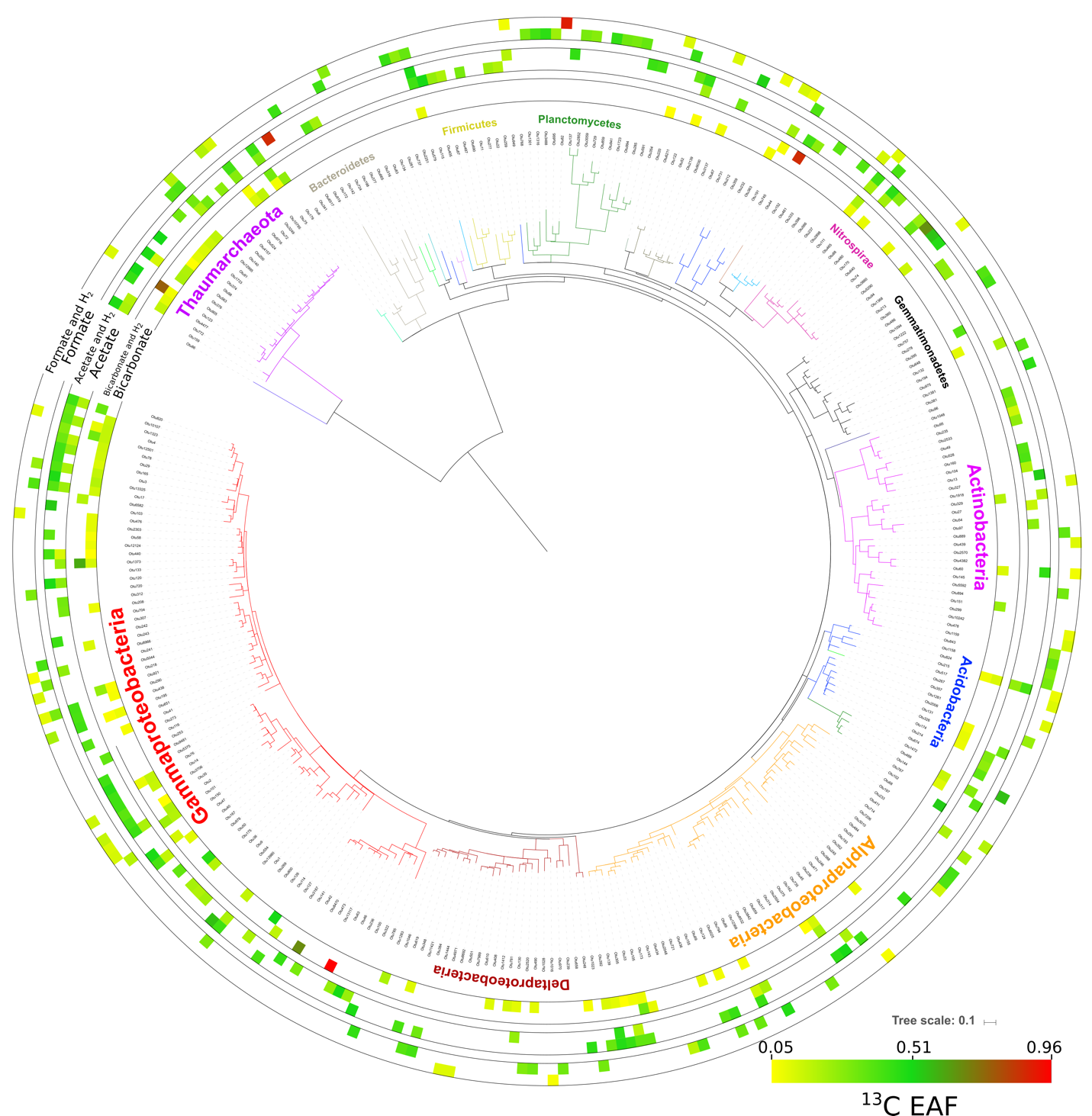

Supplement: Supplementary file 1 — Supplemental Materials [file 41396_2021_1066_MOESM1_ESM.docx]
